# Physiological Changes Associated with Aging and Immobility

**DOI:** 10.1155/2012/468469

**Published:** 2012-05-03

**Authors:** Yamni Nigam, John Knight, Sharmila Bhattacharya, Antony Bayer

**Affiliations:** ^1^College of Human and Health Science, Swansea University, Swansea SA2 8PP, UK; ^2^Biomodel Performance Laboratory, Space Biosciences Division, NASA Ames Research Center, Moffett Field, CA 94035, USA; ^3^Department of Geriatric Medicine, University Hospital Llandough, Cardiff University, Penarth CF64 2XX, UK

Aging, an inevitable and extremely complex, multifactorial process, is characterised by the progressive degeneration of organ systems and tissues. It is largely determined by genetics, and influenced by a wide range of environmental factors, such as diet, exercise, exposure to microorganisms, pollutants, and ionising radiation. This explains why two people of the same age may differ markedly in terms of both physical appearance and physiological state. Gender also plays a part and, in most developed countries, women typically outlive men by 7–10 years [1]. Recent research has also demonstrated that distant experiences such as childhood personality and education, as well as behavioural factors, also contribute to longevity [2].

It is generally accepted that the aging process falls physiologically into three groups of changes that occur with advancing age [3]. The first group encompass changes in cellular homeostatic mechanisms, for example, body temperature, blood, and extracellular fluid volumes; the second group are related to a decrease in organ mass; the third and possibly the most important group of changes, in terms of their impact, involve a decline in and loss of the functional reserve of the body's systems. Loss of these functional reserves may impair an individual's ability to cope with external challenges such as surgery or trauma. Maintaining physiological function (health) in an aging population is of prime importance not only to the well-being of the aging individual, but also from a social perspective, helping to reduce the burden on medical services and systems [4].

It has also been long established that the physiological changes associated with normal aging are mirrored during periods of immobility, such as prolonged hospital bed rest, or after a fractured limb or a fall.

It was with the above in mind and with the hope of collating research and knowledge examining the effects of normal aging and immobility that we developed the call for this special issue.

Three of the seven papers in the Issue discuss physiological changes in muscle tissue:

- age-related loss of muscle strength is considered by G. Goldspink, who pays special consideration to declining levels of Mechano Growth Factor (MGF) with age and the positive effects seen on muscle cells when this factor is externally administered;
- J. Alwood et al. describe how their study in mice shows changes to the skeletal musculature following low dose ionising gamma radiation changes which are normally seen in elderly patients prior to the onset of age-related osteoporosis;
- a quantitative review of age-related changes in strength/power and balance and the consequence of falls risk assessment is presented by U. Granacher et al.

Two papers in this special issue consider age-related cardiac function:

- G. A. Maranhao Neto et al. discuss how low levels of cardiorespiratory fitness (CRF) can be associated with health problems in elderly patients; the authors present a unique model of assessing levels of CRF, negating the use of aerobic exercise which often presents severe limitations as a test method;
- the study by S. Moodithaya and S. T. Avadhany highlights the findings that there are gender differences in age-related changes in cardiac autonomic control, suggesting that female sex hormones may play a part in cardiac autonomic modulation.

Our penultimate paper by C. N. Ross et al. explores the potential for using translational research (using a population of nonhuman primates) to determine if certain body measurements and phenotypes are associated with age or increased mortality.

Finally, R. Semprini et al. suggest a look at impaired cognition and apathy as markers for unsuccessful aging and frailty.

The papers we present here certainly, in our minds, contribute to the further understanding of the physiological changes associated with aging and highlight the continued need to develop and expand our knowledge in this important field of research.

*Yamni Nigam* *Yamni Nigam* *John Knight* *John Knight* *Sharmila Bhattacharya* *Sharmila Bhattacharya* *Antony Bayer* *Antony Bayer*
